# Machine perfusion of liver grafts: hypothermic versus normothermic versus normothermic regional perfusion

**DOI:** 10.1097/JS9.0000000000002648

**Published:** 2025-06-28

**Authors:** Damiano Patrono, Luca Del Prete, Janina Eden, Philipp Dutkowski, James V. Guarrera, Cristiano Quintini, Renato Romagnoli

**Affiliations:** a General Surgery 2U – Liver Transplant Unit, Azienda Ospedaliero Universitaria Città della Salute e della Scienza di Torino, Turin, Italy; b General Surgery and Liver Transplant Unit, IRCCS Fondazione Ca’ Granda Ospedale Maggiore Policlinico di Milano, Milan, Italy; c Translational Medicine PhD Program, University of Milan, Milan, Italy; d Department of Surgery, Section of HPB Surgery and Liver Transplantation, University of Groningen and University Medical Center Groningen, Groningen, The Netherlands; e UMCG Comprehensive Transplant Center, Groningen, The Netherlands; f Department of Visceral Surgery, University Hospital Basel, Basel, Switzerland; g Department of Surgery, Division of Transplant and HPB Surgery, Rutgers NJMS/University Hospital, Newark, NJ, USA; h Department of Liver Transplantation, Cleveland Clinic Abu Dhabi, Abu Dhabi, United Arab Emirates

**Keywords:** ischemic cholangiopathy, machine perfusion, machine perfusion indications, normothermic regional perfusion, sequential machine perfusion

## Abstract

Machine perfusion (MP) techniques, including hypothermic oxygenated perfusion (HOPE), normothermic perfusion (NMP), and normothermic regional perfusion (NRP), have emerged as strategies to optimize outcomes of extended criteria donor (ECD) livers. Despite increasing adoption, clinical indications remain uncertain. To assist transplant professionals in clinical decision making, a systematic review and meta-analysis was conducted according to PRISMA guidelines, including randomized controlled trials (RCT) and cohort studies. Outcomes of interest were ischemic cholangiopathy (IC) and graft survival. Moderator analyses explored the influence of donor, recipient, and procedural factors. Studies comparing different MP techniques and sequential approached were also reviewed qualitatively. HOPE was associated with a significant reduction in IC (RR 0.50; CI 0.31, 0.79; *P* = 0.003) and improved graft survival (RR 1.08; CI 1.05, 1.08; *P* < 0.001), with evidence supported by RCT. NMP did not significantly influence IC or graft survival. Based on retrospective studies, NRP significantly improved IC (RR 0.1, CI 0.05, 0.21; *P* < 0.0001) and graft survival (RR 1.11; CI 1.05, 1.17; *P* = 0.0001) as compared to super-rapid recovery in controlled donation after circulatory death (DCD). Sequential approaches showed promise in high-risk grafts but require further validation. Studies comparing different MP approaches are still limited. Available literature is largely heterogeneous regarding risk profile and characteristic on included donors, study designs and considered endpoints, limiting the possibility to provide clear recommendation about clinical indications. Further comparative trials and studies focusing on specific donor-recipient scenarios are necessary to refine MP utilization and optimize LT outcomes.

## Introduction

Liver transplantation (LT) is the treatment of choice for end-stage liver disease and selected forms of primary and secondary liver cancer, but it is limited by the availability of suitable donors. Utilization of liver grafts from so-called “extended criteria” donors (ECD), a heterogeneous category broadly encompassing those grafts whose utilization is associated with inferior outcomes of LT, has been one main strategy to expand donor pool. In the attempt to enhance feasibility and outcomes of LT performed with ECD liver grafts, machine perfusion (MP) techniques for organ preservation and procurement have been reintroduced in clinical practice and their adoption is increasing at a fast pace worldwide^[1-4]^. While several approaches have been described, the most adopted MP techniques are hypothermic oxygenated machine perfusion (HOPE), normothermic machine perfusion (NMP) and normothermic regional perfusion (NRP). Both HOPE and NMP are applied ex-situ after a period of cold preservation (end-ischemic approach) or throughout preservation (also referred to as machine preservation). In contrast, NRP is applied during procurement in the donor, allowing recirculation of oxygenated donor blood in the abdominal compartment (abdominal NPR, A-NRP) of in both the thorax and abdomen (thoraco-abdominal NRP, TA-NRP).

To different extent and notwithstanding some relevant nuances, three main features apply to all aforementioned machine perfusion techniques: (1) improved preservation and mitigation of ischemia-reperfusion injury as compared to static cold storage (SCS); (2) the possibility to gather data associated with liver function in the recipient (so-called viability assessment); (3) improved transplant logistics. However, ideal scenarios for their utilization may differ substantially, also depending on local availability and logistical factors. Published studies included donors and recipients with heterogeneous characteristics, complicating the process of driving practical indications from study data. Although a number of recent systematic reviews and meta-analyses have assessed the effect of the use of different techniques on clinical outcomes^[5-14]^, the confounding effect of donor and recipient factors has been poorly explored. Furthermore, most previous reviews have excluded studies reporting on the association of different techniques, which may be of value in particular settings. As a consequence, clinicians having to deal with the multifaceted interplay of donor-recipient factors may still be uncertain about the indication for MP and about the choice for one particular technique.

Based on the available literature, this review will assess the effect of HOPE, NMP and NRP on two clinically relevant and ischemia-reperfusion-related endpoints, namely the incidence of ischemic cholangiopathy and graft survival, taking into account potentially interfering donor and recipient factors. Comparative studies and studies reporting on the sequential application of different MP techniques will also be considered to define utilization settings and potential benefits. The aim is to provide transplant professionals with key concepts about indications and potential pitfalls of different MP techniques, facilitating clinical decision making in everyday clinical practice.

## Methods

A literature search was conducted on November 20th, 2024, following the PRISMA guidelines[15]. The PubMed and Web of Science databases were searched for articles published in the English language after January 1st, 2000, using the search terms “hypothermic oxygenated machine perfusion,” “normothermic machine perfusion” and “normothermic regional perfusion.” Two authors (D.P. and L.D.P.) independently performed article screening and selection, retrieval of full-text manuscripts and data extraction. Conflicts were resolved by discussion and agreement. Randomized controlled trials (RCT) and cohort studies reporting on clinical data were considered eligible for inclusion. Case reports, case series lacking a control group and review articles were excluded. When two or more articles reported duplicate data, the most recent publication reporting the outcome of interest was included. Cohort studies and RCT were assessed by the Newcastle-Ottawa scale for cohort studies[16] and the Cochrane Collaboration risk-of-bias tool[17], respectively. The manuscript is compliant with the TITAN (Transparency In The reporting of Artificial Intelligence) guidelines 2025[18]. Artificial intelligence (AI) was not used in the research and manuscript development.

Incidence of ischemic cholangiopathy and graft survival were the two outcomes used for meta-analysis. Comparator group was recipients of a graft preserved by SCS for HOPE and NMP or procured by super-rapid recovery (SRR) for NRP. An additional meta-analysis was conducted comparing LT with DCD grafts procured by NRP to LT using DBD donors. Only studies on the use of NRP in controlled DCD donors were included in the meta-analysis. Variables considered for the moderator analysis were donor age, cold ischemia time, recipient age and MELD, percentage of DCD graft in the intervention group (HOPE and NMP), use of single versus dual HOPE (HOPE), use of back-to-base versus upfront NMP (NMP) and functional warm ischemia time (NRP). Clinical studies reporting on the sequential use of different MP techniques (e.g., NRP + HOPE or D-HOPE-controlled oxygenated rewarming (COR)-NMP) or comparing different MP approaches were retrieved but not included in the meta-analyses.

**HIGHLIGHTS**

- A literature search was performed to assess the effect of hypothermic oxygenated machine perfusion (HOPE), normothermic machine perfusion (NMP) and normothermic regional perfusion (NRP) on the incidence of ischemic cholangiopathy and graft survival in liver transplantation.
- Level 1 evidence supports the used of HOPE to reduce incidence of ischemic cholangiopathy and improve graft survival in recipients of livers from donor whose death was determined by both neurological (DBD) and circulatory (DCD) criteria.
- Retrospective cohort studies support the beneficial effect of NRP on ischemic cholangiopathy and graft survival in DCD liver transplantation, while the main advantage of NMP is related to the possibility to assess liver viability and maximize donor pool utilization.
- Sequential application of different machine perfusion techniques may be of value in high-risk donors
- Limitations of the available literature include the inclusion of donors with a heterogeneous risk profile, study design and definitions of considered endpoints, limiting the possibility to provide definitive recommendation about clinical indications.

To conduct meta-analyses, the metafor package in R was used^[19,20]^. Data were first extracted from relevant studies, including effect sizes (risk ratio, RR) and their corresponding variances or standard errors. The effect sizes were transformed as necessary to ensure compatibility across studies. We fitted a random-effects model to account for heterogeneity among studies, estimating the overall effect size and its confidence interval with the rma() function. Heterogeneity was assessed using the Q-statistic, I^2^-index, and τ^2^. Funnel plots and Egger’s test were used to examine publication bias. Moderator analyses were performed to rule out a potential effect of donor, recipient or procedural variables on the observed effect sizes. Influential case diagnostics was performed using Baujat plots and by the influence() function. Sensitivity analyses were conducted by iteratively removing studies to evaluate the robustness of results. Significance level was set at 0.05. All analyses and data visualization were performed in R (version 4.4.1, R Foundation for Statistical Computing, Vienna, Austria). PRISMA flow diagram was generated using a free online-available shiny app (https://estech.shinyapps.io/prisma_flowdiagram/)[21].

## Results

### Hypothermic oxygenated machine perfusion (HOPE/D-HOPE)

Eleven articles were included in the meta-analysis, including 6 RCT^[22–28]^ and 5 cohort studies^[29–33]^ (Table 1), reporting 714 HOPE LT (DCD, n = 148) and 1392 SCS LT. Weighted median (IQR) donor age, cold ischemia time, recipient age and MELD were 55 (53, 72) years, 5.1 (4.4, 6.1) hours, 58 (57, 60) years and 14.2 (12.6, 15.5).

**Table 1:** Literature quality assessment

				Cochrane RoB2							NOS			
Author	Year	Design	n^a^	Outcome	Randomization process	Deviation from intended interventions	Missing outcome data	Measurement of the outcome	Selection of the reported results	Overall	Selection	Comparability	Outcome	Total
**HOPE versus SCS**														
Morawski *et al*^b^	2024	RCT	26	IC	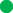	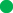	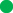	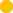	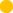	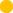				
				GS	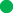	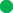	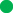	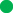	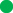	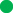				
Czigany *et al*	2024	RCT	23	GS	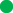	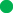	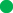	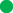	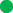	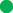				
Panayotova *et al*	2023	RCT	63	IC	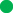	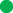	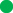	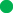	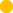	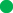				
				GS	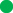	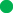	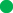	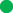	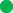	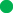				
Schlegel *et al*	2023	RCT	85	GS	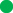	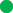	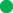	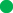	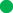	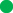				
Ravaioli *et al*	2022	RCT	55	GS	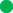	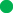	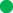	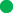	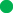	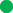				
van Rijn *et al*	2021	RCT	78	IC	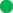	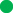	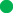	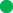	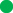	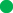				
				GS	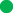	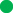	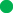	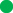	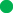	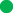				
Pereyra *et al* (Single-HOPE)	2024	Cohort	76								3	2	3	8
Pereyra *et al* (Dual-HOPE)	2024	Cohort	102								3	2	3	8
Patrono *et al*	2022	Cohort	121								4	2	3	9
Rayar *et al*	2020	Cohort	25								4	2	2	8
Schlegel *et al*	2018	Cohort	50								4	2	3	9
van Rijn *et al*	2017	Cohort	10								4	2	2	8
**NMP versus SCS**														
Chapman *et al*	2023	RCT	136	IC	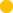	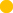	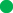	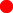	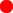	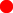				
				GS	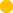	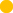	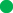	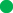	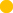	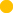				
Markmann *et al*	2022	RCT	151	IC	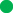	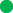	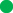	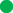	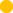	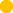				
Ghinolfi *et al*	2019	RCT	10	IC	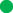	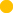	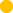	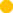	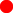	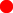				
				GS	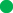	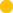	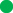	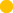	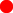	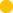				
Nasralla *et al*	2018	RCT	121	IC	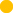	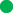	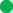	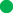	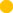	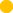				
				GS	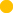	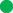	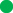	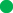	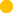	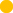				
Hefler *et al*	2023	Cohort	79								3	2	3	8
Gaurav *et al*	2022	Cohort	67								4	2	2	8
Fodor *et al*	2021	Cohort	59								4	2	2	8
Bral *et al*	2017	Cohort	9								3	1	2	6
Ravikumar *et al*	2016	Cohort	20								4	2	3	9
**NRP versus SRR**														
Brubaker *et al*	2024	Cohort	106								4	2	3	9
Gaurav *et al*	2022	Cohort	69								4	1	2	7
Hessheimer *et al*	2022	Cohort	545								4	1	2	7
Muñoz *et al*	2020	Cohort	23								3	2	2	7
Watson *et al*	2019	Cohort	43								4	3	2	9
**NRP versus DBD**														
Campo *et al*	2023	Cohort	177								4	2	3	9
Rodriguez *et al*	2022	Cohort	39								2	1	2	5
Viguera *et al*	2021	Cohort	62								3	2	2	7
Ruiz *et al*	2021	Cohort	100								4	2	3	9
Savier *et al*	2020	Cohort	50								4	2	3	9
Rodríguez-Sanjuán *et al*	2019	Cohort	11								2	2	2	6

DBD, donation after brain death; GS, graft survival; HOPE, hypothermic oxygenated machine perfusion; IC, ischemic cholangiopathy; NMP, normothermic machine perfusion; NOS, Newcastle-Ottawa scale; NRP, normothermic regional perfusion; RoB2,Cohcrane risk of bias tool 2; SCS, static cold storage; SRR, super-rapid recovery.

^a^ number in the intervention group.

^b^ The study by Morawski *et al* reported long-term outcome data, including graft survival and incidence of ischemic cholangiopathy, from the Grat *et al* trial, which was therefore not included in the meta-analysis. Color code for the Cochrane risk of bias 2 tool; 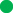, low risk; 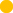, some concerns; 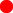, high risk.

The first published RCT comparing HOPE to SCS was the DHOPE-DCD trial by van Rijn *et al*[22], which demonstrated a significant reduction of symptomatic non-anastomotic strictures (6% vs. 18%, *P* = 0.03) and of the number of required treatments (5 vs. 22) at the 6-month follow-up in recipients of a DCD grafts. Notably, two patients underwent re-LT in the control group versus none in the intervention group. Two positive trials including grafts from DBD donors followed, namely the HOPE ECD-DBD trial by Czigany *et al*[23] and the study by Ravaioli *et al*[24]. Both trials met their primary endpoint, which was peak ALT levels in the first 7 days after LT (418 vs. 796, *P* = 0.03) and incidence of early allograft dysfunction (EAD)[34] (13% vs. 35%, *P* = 0.007). Long-term follow-up data from the HOPE ECD-DBD trial have confirmed a reduction of late-onset morbidity and improved long-term graft survival[35]. The trials by Schlegel *et al*[27] and Grat *et al*[25] did not meet their primary endpoints, which were incidence of Clavien–Dindo grade ≥III complications within 1 year from LT and model for early allograft failure[36], respectively. In both studies, however, post-hoc analyses suggested superior graft preservation by HOPE, determining an improved graft function in recipients of ECD DBD grafts and a reduction of liver-related severe (grade ≥ IIIb) complications and graft loss. Morawski *et al*[26] recently reported 2-year follow-up data of the Grat *et al* trial[25], confirming that benefits of HOPE were restricted to recipients of high-risk grafts. Lastly, the study by Panayotova *et al*[28], which was designed as a non-inferiority trial, showed an EAD incidence (primary endpoint) of 11.1% versus 16.4% in the HOPE and SCS group, respectively. This trial, which employed a transportable MP device in which perfusate is pre-oxygenated, also showed a reduction of the risk of graft failure as assessed by the L-GrAFT_7_ model (3.4% vs. 4.5%, *P* = 0.024).

**Figure 1.**
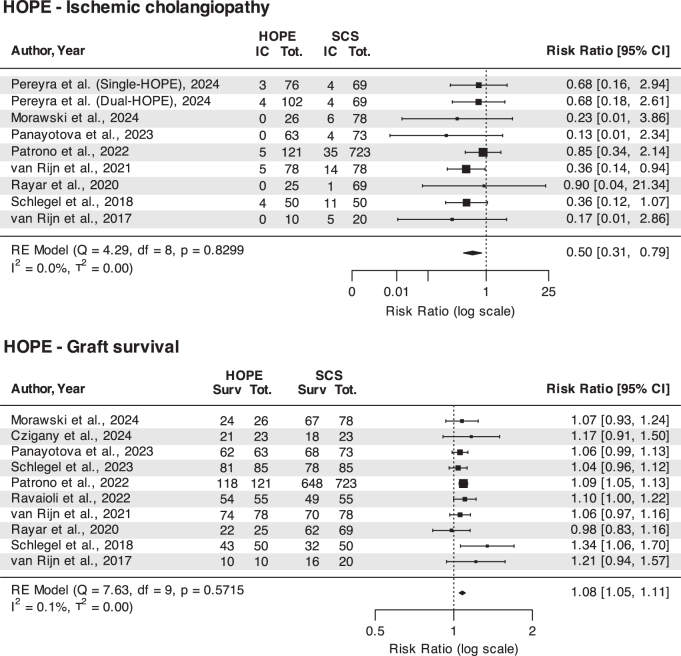
Foster plots illustrating the effect of hypothermic oxygenated machine perfusion (HOPE) on the incidence of ischemic cholangiopathy and graft survival in liver transplantation.

Results of the meta-analysis are shown in Fig. 1. The study by Pereyra *et al*[29], which reported outcome data separately according to the use of single versus dual HOPE, was considered as two distinct studies. Eight studies reported data about the incidence of IC and ten about graft survival. In keeping with previous meta-analyses^[5-8]^, the use of HOPE was associated with a significant reduction of IC (RR 0.50; CI 0.31, 0.79; *P* = 0.003) and improved graft survival (RR 1.08; CI 1.05, 1.08; *P* < 0.001), with low heterogeneity across included studies and no evidence of publication bias. Moderator analysis did not show any significant effect of the percentage of DCD included in the study, use of single versus dual HOPE, donor age, cold ischemia time, recipient age and MELD. Influential case diagnostics showed a potential influence on the effect size by the inclusion of the Patrono *et al*[30] study. However, the sensitivity analysis performed removing this study confirmed the beneficial effect of HOPE on IC and graft survival. To inform donor-recipient matching and help refine clinical indications, a subset analysis was performed with studies including only DCD and DBD donors, respectively. In DCD LT (3 studies including 138 HOPE and 148 SCS transplants), the protective effect of HOPE on IC was confirmed, whereas the positive effect on graft survival was borderline significant (RR 1.16; CI 0.99, 1.15; *P* = 0.059). When the analysis was restricted to studies including only DBD donors (6 studies including 335 HOPE and 1033 SCS transplants) the positive effect on graft survival persisted (RR 1.08; CI 1.05, 1.11; *P* < 0.001), whereas the protective effect on IC was not significant. All PRISMA flow diagrams, Funnel plots, moderator analyses, influential case diagnostics and sensitivity analyses are presented as Supplementary Digital Content (available at: http://links.lww.com/JS9/E526).

Benefits of HOPE not captured by the meta-analysis include the possibility to extend preservation time and to test liver viability during MP. At least two large retrospective studies^[37,38]^ and a prospective trial from the Groningen group[39] have shown that HOPE can safely extend total preservation time up to 24 hours, with the potential to facilitate transplant logistics and avoid nighttime LT. Concerning the possibility to perform a viability assessment during HOPE, which has for long been considered an inherent limitation of the technique, recent studies have shown that perfusate levels of flavin mononucleotide (FMN), a cofactor of mitochondrial complex I, are associated with the degree of injury and function of the liver post-LT and correlate with clinical outcomes^[40–42]^. Although perfusate FMN during HOPE has been recently validated as a marker of mitochondrial injury in a multicenter study[43], its use as a viability marker is still not widespread.

HOPE has been widely implemented in Europe and real-world long-term outcome data have recently been published in the HOPE-REAL study[44], which reported excellent 5-year graft survival for both DBD (91%) and DCD (81%) grafts, with a low incidence of IC at 2-year follow-up (DBD: 2.5%; DCD: 11.5%).

### Normothermic machine perfusion (NMP)

Literature search retrieved 9 articles (RCT, n = 4; cohort studies, n = 5) that were included in the meta-analysis^[45–53]^, reporting on 652 NMP LT (DCD, n = 212) and 993 SCS LT (Table 1). Weighted medians (IQR) of variables retrieved as possible moderators were as follows: donor age 52 (44, 55) years; cold ischemia time 2.8 (2.1, 5.2) hours; recipient age 58 (57, 59) years; recipient MELD 15.5 (13, 17.8).

The study by Narsalla *et al*[47], which was the first RCT on MP being published, included 222 LT (NMP, n = 121; SCS, n = 1010) and met its primary endpoint demonstrating a significant reduction of AST peak during the first 7 days after LT (with the first value measured from 12 to 24 hours after LT) from 964 to 488 IU/L (*P* < 0.001). This study also showed a lower discard rate in the NMP arm (11.7% vs. 24.1%), but comparable rate of IC and graft survival. The study by Ghinolfi *et al*[48], which is so far the only RCT having investigated NMP used back-to-base and included DBD grafts from ≥70-year-old donors (n = 10 in each arm), did not demonstrate an advantage in terms of graft and patient survival (primary endpoints), although electron microscopy suggested better histological preservation in the NMP arm. Two large United States-based RCT followed in 2022 and 2023. The study by Markmann *et al*[51] compared 151 LT using NMP with 142 using SCS and demonstrated a significant reduction of EAD (18% vs. 31%, *P* = 0.01) in the treatment group. Besides showing a lower discard rate of DCD grafts (49% vs. 74%) in the treatment arm, this study was also the first suggesting a reduction in the incidence of IC (2.6% vs. 9.9% at 12 months, *P* = 0.02). In contrast, the study by Chapman *et al*[52], which compared 136 LT using NMP to 130 using SCS, did not show a significant reduction of EAD (20.6% vs. 26.7%, *P* = 0.27). This discrepancy was attributed to the prevalent inclusion of low-risk livers. Indeed, the protective effect of NMP emerged when the analysis was restricted with higher-risk and DCD livers. Ischemia-free LT (IFLT) is a particular variant of NMP by which SCS can be completely avoided, starting NMP in the donor until graft reperfusion into the recipient. Guo *et al*[54] conducted a RCT comparing 32 LT performed by IFLT with 33 using SCS, showing lower EAD, post-reperfusion syndrome, as well as lower incidence of IC and of surgical complication. Due to the peculiarities of the technique this study was not included in the meta-analysis.

**Figure 2.**
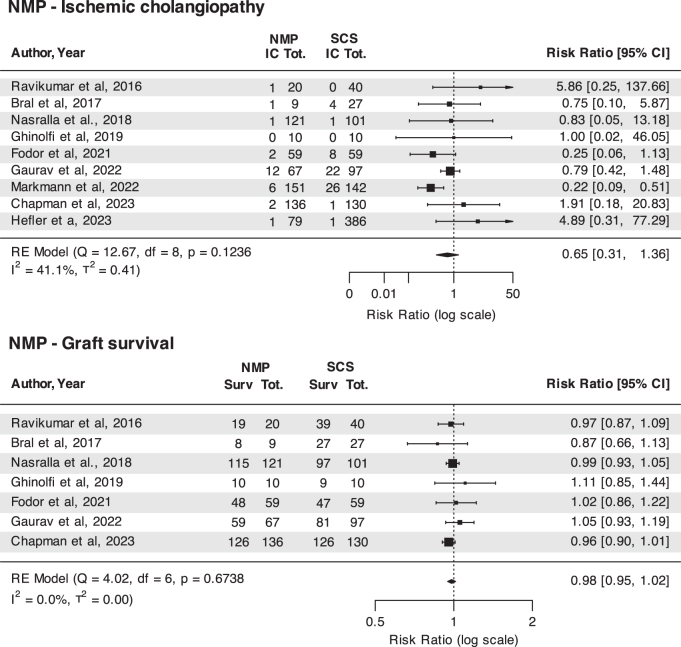
Foster plots illustrating the effect of normothermic machine perfusion (NMP) on the incidence of ischemic cholangiopathy and graft survival in liver transplantation.

Results of the meta-analysis (Fig. 2) did not show a significant effect of NMP on the incidence of IC and graft survival after LT, with low heterogeneity across included studies and no evidence of publication bias. Moderator analysis showed an apparent protective effect of higher recipient MELD on the incidence of ischemic cholangiopathy. However, this finding is likely explained by the fact that the study by Markmann *et al*[51], which was the only one showing a protective effect of NMP toward IC, included patients with higher median MELD (29) as compared to the other included studies (range: 12-18). Subset analyses by donation modality were complicated by the fact that most studies did not report separate outcomes for DCD and DBD donors or did not report data on the outcome of interest (e.g., ischemic cholangiopathy). Therefore, only 3 studies focusing on DCD donors and 2 on DBD donors were included. Subset analyses did not show any significant effect of NMP on IC rate or graft survival (Supplementary Digital Content, available at: http://links.lww.com/JS9/E526).

A feature not captured by the above meta-analysis is the use of NMP for liver viability assessment. Given the possibility to observe a metabolically active organ in an isolated environment, NMP has been extensively used to assess graft viability and to enable transplantation of organs that were initially discarded. Despite some differences in evaluation protocols^[55,56]^, the discussion of which is beyond the scope of this review, use of NMP in this setting has allowed recovering 47% to 100%^[57–66]^ of tested livers. A recent study by Wehrle *et al*[67] analyzed the impact of the implementation of acuity circles and NMP at two large centers in the US, where NMP is currently the only MP technique approved for clinical use. NMP adoption resulted in a reduction of waiting time and an increased number of LT performed, which was mainly due to the use of donors with higher-risk profile with a 95% utilization rate. Notably, use of higher-risk donors did not worsen LT outcomes and shorter waiting time also resulted in a reduction of costs.

Similarly to HOPE, NMP can and is being used to facilitate transplant logistics. Commercially available devices can extend NMP time up to 24 hours, leading to the possibility to transform liver transplantation from an urgent, time-sensitive operation to a more controlled and semi-elective procedure. In the same study by Wehrle *et al*[67] total preservation time was 6 hours when SCS was used versus 16 in case of NMP, confirming its positive effect on transplant logistics.

### Normothermic regional perfusion (NRP)

We identified 5 cohort studies^[50,68–71]^ comparing NRP to super-rapid recovery (SRR) in controlled DCD donors that were included in the meta-analysis (Table 1). No RCT has compared NRP to SRR. Of the available studies, 4 were retrospective multicenter and 1 retrospective single-center, including 786 livers procured using NRP and 700 procured by SRR. Five studies reported data on the incidence of IC and 4 on graft survival. Weighted medians (IQR) of the variables retrieved as potential effect moderators were: donor age 53 (46, 56) years; functional warm ischemia time (fWIT) 12 (12, 15) minutes; cold ischemia time 5.1 (4.9, 5.3) hours; recipient age 58 (57, 59) years; recipient MELD 12 (12, 12).

The largest included study was that by Hessheimer *et al*[70], which compared outcomes of livers from Maastricht category III DCD donors procured by abdominal NRP (n = 545) versus SRR (n = 258). By adjusting risk estimates of each outcome by several donor, recipients and procedural variables, this study showed an inferior rate of IC and graft loss when NRP is used, suggesting that longer cold ischemia time and redo-LT are risk factors for graft loss.

**Figure 3.**
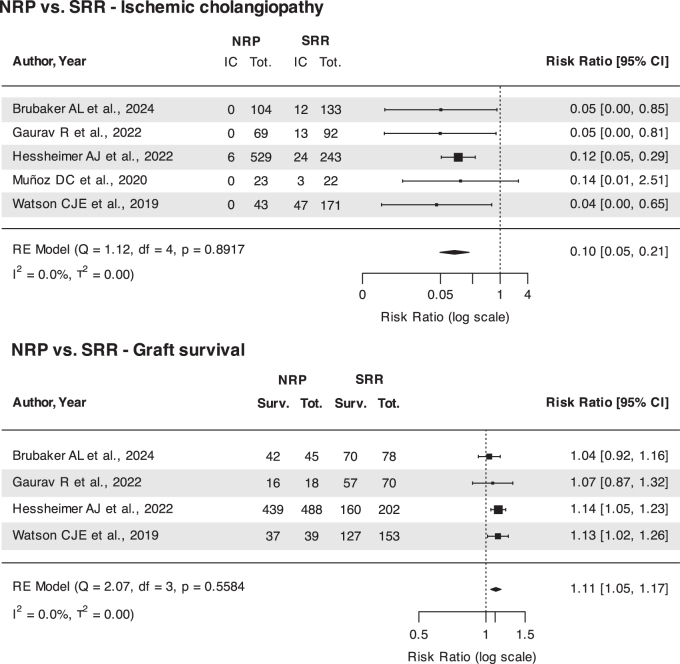
Foster plots illustrating the effect of normothermic regional perfusion (NRP) on the incidence of ischemic cholangiopathy and graft survival in liver transplantation.

Meta-analysis (Fig. 3) showed that use of NRP as compared to SRR reduced the incidence of IC (RR 0.1, CI 0.05, 0.21; *P* < 0.0001) and improved graft survival (RR 1.11; CI 1.05, 1.17; *P* = 0.0001), with low heterogeneity across included studies (Fig. 3). Moderator analysis did not show a significant effect on results by donor age, warm ischemia and cold ischemia time, recipient age and MELD. Influential case diagnostics showed a possible influence of the study by Hessheimer *et al*[70] on IC, and of the studies by Brubaker *et al*[71] and Hessheimer *et al*[70] on graft survival. However, sensitivity analyses performed removing these studies did not significantly change the results (Supplementary Digital Content, available at: http://links.lww.com/JS9/E526) and the protective effect of NRP persisted.

We performed an additional meta-analysis including 6 studies^[72–77]^ comparing outcomes of DCD LT using NRP (n = 439) with matched DBD LT (n = 2215). Five studies reported data on the incidence of IC and 5 on graft survival. The meta-analysis (Supplementary Digital Content, available at: http://links.lww.com/JS9/E526) showed that use of NRP in the setting of controlled DCD LT allowed achieving comparable outcomes in terms of IC (RR 0.51; CI 0.63, 1.66; *P* = 0.38) and suggested a positive effect on graft survival (RR 0.04; CI 0.01, 0.08; *P* = 0.02). However, influential case diagnostics showed a possible influence due to the inclusion of the studies by Ruiz *et al*[74] and Campo-Canaveral de la Cruz *et al*[77] and the sensitivity analysis performed after removing these two studies showed comparable survival (RR 0.04; CI −0.03, 0.11; *P* = 0.23).

Besides improving LT outcomes, use of NRP has been linked to improved DCD organ utilization^[78,79]^, also in the setting of uncontrolled DCD donation[80]. This may at least partially be attributed to the possibility to assess liver injury and function during NRP, thereby discarding those livers with an injury profile that is perceived as prohibitive. Viability criteria during NRP are largely heterogeneous, but are most commonly based on pump parameters, macroscopic evaluation of the graft and abdominal viscera, liver histology, transaminases, and lactate clearance[81]. As compared to viability assessment during ex-situ MP, assessment during NRP may suffer from local factors that are difficult to control, like the amount of fluids and packed red blood cells infused, areas of bowel necrosis, and spillage of blood from ischemic areas (thorax, limbs)[50].

Regarding transplant logistics, the benefit of NRP as compared to SRR is mainly due to the possibility to perform a procurement in a more relaxed fashion, performing a careful evaluation of abdominal viscera and donor hepatectomy, reducing the likelihood of procurement-related injuries. In this regard, it is significant that in the paper by Hessheimer *et al*[70] 13/775 (2%) of livers procured by NRP were discarded due to a previously undiagnosed malignancy versus none (0/390) in the SRR group.

### Comparative studies

Some retrospective studies have compared different MP approaches in controlled DCD donors. Muller *et al*[82] compared outcomes of livers procured using NRP and transplanted at 6 French centers from those of end-ischemic HOPE at the Zurich center. Donors in the HOPE group were older, had higher BMI, and fWIT was longer (31 vs. 22 minutes, *P* < 0.001). Cold ischemia time was shorter (4 vs. 5.7 hours, *P* < 0.001) in the HOPE group, whereas total preservation time was longer (6.4 vs. 5.7 hours, *P* < 0.001). Despite higher utilization rate in the HOPE group (81% vs. 63%) incidence of IC and graft survival were comparable. Mohkam *et al*[83] compared LT after in-situ NRP at 6 French centers (n = 157) with 40 DCD LT using ex-situ NMP from the COPE trial[47], using propensity score matching to overcome allocation bias. Among the donors that proceeded to asystole, utilization rate was higher in NMP group (85% vs. 70%, *P* = 0.052). With a median follow-up of 23 months, incidence of IC in the NMP and NRP group (2.9% vs. 1.5%, *P* = 1) and 2-year graft survival (88.2% vs. 89.4%, *P* = 0.52) were comparable. Lastly, Gaurav *et al*[50] compared outcomes of DCD livers preserved by SCS (n = 97) versus NMP (n = 67) versus NRP (n = 69) and transplanted at the Roy Calne Transplant Unit (Addenbrooke’s Hospital, Cambridge, UK). Overall, while NRP was associated with better graft survival and IC rate as compared to SCS, the same was not observed for back-to-base NMP. IC rate and 1-year graft survival were 6% versus 19% and 93% versus 88% in the NRP and NMP group, respectively.

### Sequential approaches

Based on pre-clinical studies^[84,85]^, the combination of HOPE followed by a period of controlled oxygenated rewarming (COR) and NMP (HOPE-COR-NMP) has been advocated to combine the positive effects of HOPE on mitochondrial metabolism and the possibility to test liver viability during NMP. Clinical series (Table 2) have been reported by the Groningen^[86–89]^ and Cleveland[90] groups for a total of 122 cases, of which 82 were transplanted (use rate: 68.3%). This approach has primarily been used for DCD livers with unfavorable donor profile, as reflected for example by a median donor age of 66 (61, 71) in the Groningen series. Outcomes of LT performed using HOPE-COR-NMP have been compared to contemporary DBD LT[89] or to end-ischemic NMP alone[90], with similar results. Based on the available literature it is unclear whether HOPE-COR-NMP offers a significant advantage over NMP alone.

**Table 2:** Articles reporting on the sequential use of HOPE-COR-NMP

First author	Year	Design	n	DCD (%)	Use rate	Donor age	Functional WIT (min)	SCS (min)	IC	1-year graft survival
de Vries *et al*	2018	Retrospective, single-center	7	100.0	5/7 (71.4%)	52 (46–62)	23–35	289	0/5 (0%)	na
Van Leeuwen	2019	Retrospective, single-center	16	68.8	11/16 (68.8%)	63 (52–72)	na	270	1/11 (9.1%)	100%
Liu	2022	Retrospective, single-center	17	52.9	13/17 (76.5%)	52 (39–61)	na	351	2/13 (15.4%)	100%
Van Leeuwen	2022	Retrospective, single-center	54	61.1	34/54 (63%)	66 (56–70)	29	271	1/34 (2.9%)	94%
Van Leeuwen	2023	Retrospective, single-center	105	64.8	69/105 (65.7%)	66 (61–71)	30	na	2/69 (2.9%)	93%

Data are presented as count (%) or median (IQR).

COR, controlled oxygenated rewarming; HOPE, hypothermic oxygenated machine perfusion; IC, ischemic cholangiopathy; na, not available; NMP, normothermic machine perfusion; SCS, static cold storage; WIT, warm ischemia time.

Pushed by local regulations determining prolonged warm ischemia time in DCD donors, Italian centers have pioneered the combination of in-situ NRP followed by ex-situ MP, which has been HOPE in the majority of cases (Table 3)^[91–99]^. Published series are characterized by fWIT in controlled DCD donors of 36–45 minutes and median donor age ranging from 49 to 82 years, with a preferential allocation to low-MELD (range: 8-11) patients. Despite the high-risk donor profile, reported outcomes have been characterized by an IC rate of 0–5% and 1-year graft survival consistently >90% for livers from controlled DCD donors, in line with European series. In The Netherlands, a similar approach was reported by Schurink *et al*[100], who used NRP to enable successful transplantation of 20 DCD grafts declined by all centers in the Eurotransplant region. In this series HOPE was used in 25% of grafts and 1-year IC rate and graft survival were 11% and 90%, respectively. In the multicenter study by Schlegel *et al*[101] defining benchmark outcomes after DCD LT, LT performed using high-risk liver donors (defined as those with total donor WIT >30 minutes or asystolic WIT >15 minutes) preserved by NRP + HOPE (n = 63) showed IC rate and 1-year graft survival of 3.2% and 88.9%, respectively, in line with those of benchmark cases (n = 1012).

**Table 3:** Articles reporting on the sequential use of NRP + ex-situ MP

First author	Year	Design	Approach	DCD type	n	Use rate	Donor age	Functional WIT	MELD	IC	Graft survival	Other endpoints
Torri	2024	Prospective; single-center	NRP + NMP (n = 5); NRP + D-HOPE (n = 6)	Cat.3	11	11/17 (65%)	82 (78–85)	36 (34–43)	10 (9–12)	0/11 (0%)	100%^a^	PNF: 0/11 (0%) EAD: 2/11 (18%)
Fallani	2023	Retrospective; single-center	NRP + HOPE (n = 26)	Cat.3	26	26/30 (87%)	75 (64–78)	40 (38–48)	10 (8–14)	0/26 (0%)	93%^b^	PNF: 1/26 (4%) EAD 3/26 (12%)
Ghinolfi	2024	Retrospective; multicenter	NRP (n = 2) NRP + NMP (n = 10) NRP + D-HOPE (n = 28)	Cat.2	40	40/77 (52%)	49 (45–58)	140 (123–165)	10 (8-14)	4/40 (10%)	75%^b^	PNF: 5/40 (12%) EAD: 17/40 (42%)
			NRP (n = 1) NRP + NMP (n = 5) NRP + D-HOPE (n = 53)	Cat.3	59	59/76 (78%)	57 (49–63)	45 (38–81)	11 (8–16)	2/59 (3.4%)	90%^b^	PNF: 3/59 (5.1%) EAD: 13/59 (22%)
Patrono	2022	Retrospective; single-center	NRP + D-HOPE (n = 20)	Cat.3	20	22/37 (59.5%)	60 (55–61)	43 (35–46)	10 (9–14)	2 (5%)	90%^b^	PNF: 0/20 (0%) EAD: 1/20 (5%)
Dondossola	2021	Retrospective; multicenter	NRP + D-HOPE (n = 22)	Cat.2 (n = 3) Cat.3 (n = 19)	22		56 (48–59)	40 (33–51)	10 (9–12)	0/22 (0%)	91%^a^	PNF: 1/22 (4%) EAD: 5/22 (22%)
De Carlis	2021	Retrospective; multicenter	NRP (n = 7) NRP + D-HOPE (n = 37)	Cat.3	44	44/51 (86.5%)	59 (34–69)	40 (20–80)^d^	9 (6–25)	1/44 (2%)	92%^c^	PNF: 2/44 (5%)
Ghinolfi	2021	Retrospective; multicenter	NRP + NMP (n = 7) NRP + D-HOPE (n = 11)	Cat.2 (n = 11) Cat.3 (n = 9)	18	18/34 (53%)	53 (43–57)	Cat.2: 150 (140–164) Cat.3: 52 (47–74)	11 (9–16)	1/18 (5.5%)	94%^a^	PNF: 0/18 (0%) EAD: 5/18 (28%)
Olivieri	2019	Retrospective; single-center	NRP + D-HOPE (n = 10)	Cat.2 (n = 2) Cat.3 (n = 14)	10	10/16 (62.5%)	56 (35–67)	36 (34–40)	8 (7–9)	0/10 (0%)	100%^a^	PNF: 0/10 (0%)
De Carlis	2018	Retrospective; single-center	NRP + D-HOPE (n = 20)	Cat.2 (n = 14) Cat.3 (n = 6)	20	20/25 (80%)	51 (46–61)	125 (72–143)^e^	10 (8–13)	2/17 (11.8%)	85%^b^	PNF: 2/20 (10%) EAD: 4/20 (20%)

DCD, donation after determination of death by circulatory criteria; (D-)HOPE, (dual) hypothermic oxygenated machine perfusion; EAD, early allograft dysfunction; IC, ischemic cholangiopathy; MELD, model for end-stage liver disease; MP, machine perfusion; NMP, normothermic regional perfusion; NRP, normothermic regional perfusion; PNF, primary non-function.

^a^ 6-month graft survival

^b^ 12-month graft survival

^c^ 2-year graft survival

^d^ Minimum and maximum values are reported between brackets

^e^ Pooled warm ischemia time were reported for both Cat. 2 and Cat. 3 DCD donors.

## Discussion

Results of this meta-analysis show that HOPE/D-HOPE is the only MP technique for which level 1 evidence supports a beneficial effect on IC and graft survival in both DBD LT and controlled DCD LT. When the meta-analysis was restricted to studies including only DCD donors, the reduction of IC rate was confirmed, whereas the positive effect on graft survival was borderline significant, likely due to the low number of patients included in these studies. In DBD LT setting, the meta-analysis showed that HOPE was associated with improved graft survival, whereas the protective effect on IC was not significant, a finding that can be explained by the lower incidence of IC in the DBD setting. The moderator analysis, however, showed no significant effect of the percentage of DCD grafts included in each study on both considered outcomes, suggesting that HOPE is equally effective in both DCD and DBD grafts. Although a similar effect was observed for NRP in the setting of DCD LT, this was based on cohort studies, as no RCT has compared NRP to SRR. Furthermore, use of NRP in DCD LT was associated with similar incidence of IC and graft survival as compared with DBD LT. We could not find evidence of a beneficial effect of NMP on IC rate and graft survival. These results confirm those of previous meta-analyses^[5-11]^. Some limitations of the available literature, however, should be acknowledged.

Concerning IC, some studies suffered from limited follow-up or from poor definition of IC. In the trial by van Rijn *et al*[22] the incidence of radiological non-anastomotic strictures at 6-month follow-up was comparable between study groups and a longer follow-up would have been required to gather a more complete picture of the incidence of symptomatic IC. In the trial by Markmann *et al*[51] IC was poorly defined, seemingly including also anastomotic stricture. Furthermore, a constantly overlooked aspect of most literature is the severity of IC, which is associated with prognosis and could inform more than raw incidence[102].

Some studies failed to demonstrate a clinical benefit of MP likely due to study design. In the HOPE domain, the RCTs by Grat *et al*[25] and Schlegel *et al*[27] failed to meet their primary endpoint but showed a benefit in terms of liver-related severe complications or when the analysis was restricted to recipients of higher-risk livers. In these trials, HOPE was not used exclusively in ECD livers and recipients were rather unselected. The lack of effect in these trials was likely due to the good outcomes in the control group of livers preserved by SCS and by the confounding effect of recipient factors. The same limitations in design apply to two large NMP trials, namely those by Nasralla *et al*[47] and Chapman *et al*[52], which did not include specifically ECD livers, making more difficult to demonstrate a positive effect in the intervention group.

Besides limitations in study design, MP techniques were applied by heterogeneous protocols. Main differences included single versus dual cannulation of inflow vessels in HOPE and surgical versus percutaneous cannulation in NRP. With regards to NMP, it should be noted that while in RCTs NMP was mainly used by a device-to-donor approach (with the exception of the trial by Ghinolfi *et al*[48]), the end-ischemic (back-to-base) approach was predominant in cohort studies, especially more recent ones. The difference in initial cold ischemia time and consequent ischemia-reperfusion injury may vary substantially between the two approaches and influence outcomes. Although our moderator analysis suggests that the impact of these differences in MP protocols is not substantial in a pooled analysis, these might be relevant in specific high-risk scenarios.

With regard to the characteristics of included grafts, these were highly heterogeneous, with grafts suffering from moderate or severe steatosis constantly underrepresented for any MP technique. Analysis of donor and recipient variables showed that overlapping of different donor risk factors was generally avoided (e.g., in the van Rijn *et al* trial[22] donor age was limited to ≤60 years) and that livers treated with MP were preferentially allocated to low-risk recipients. This was particularly evident for studies on NRP, in which median donor age, cold ischemia time and recipient MELD were 52 years, 5.1 hours and 12, respectively. Thus, findings from the literature should be applied with caution in everyday clinical practice, especially when dealing with complex recipients. It is significant that in the study by Hessheimer *et al*[70] clinical outcomes were negatively impacted by prolonged cold ischemia time and redo-LT, confirming the importance of procedural and recipient variables also when MP techniques are applied. This concept should also inform recipient inclusion criteria in future MP trials, which suffer from an apparently unsolvable dilemma. Indeed, while restricting inclusion to low-risk patients may reduce confounding due to recipient comorbidities and reduce the risk of negative results, this would also make the study poorly reflective of everyday practice[103].

To deal with higher-risk donors, sequential approaches have been explored. Both D-HOPE-COR-NMP and sequential NRP + MP have been applied mostly to optimize outcomes and enable viability assessment of high-risk DCD livers, characterized by older age and prolonged WIT. While results are encouraging, the number of included patients is still limited, and studies lack an adequate comparator group to draw definitive conclusions. A limitation that applies to most studies on MP, and in particular to those in which NMP has been utilized for viability assessment, is a more accurate definition of included ECD donors. Ideally, ECD donors should be subclassified based on factors like donor age, cold ischemia time, donor type (DCD vs. DBD) and steatosis to investigate which MP technique would be more beneficial in each subset.

When comparing different MP approaches, limited conclusions can be drawn from the available comparative studies^[50,82,83]^. Ideally, only studies comparing different MP techniques head-to-head can define the advantages and disadvantages of each approach in different settings. At the present, there are two ongoing randomized multicenter trials comparing HOPE versus NMP, both applied by an end-ischemic approach. The HOPE-NMP trial (NCT04644744), currently recruiting, is a three-arm study comparing HOPE (n = 85), NMP (n = 85) and SCS (n = 43) in recipients of ECD grafts with 90-day comprehensive complication index[104] as a primary endpoint. The DCD-Net study (NCT04744389) will compare HOPE versus NMP, both performed after NRP, in the setting of DCD donation with prolonged warm ischemia time. 6-month graft survival and incidence of IC are the primary endpoints. The study has completed recruiting and results are expected soon. Hopefully, these studies will help refine indications and benefits of different MP approaches.

Notwithstanding the limitations of the available literature that preclude making definitive recommendations, both HOPE and NRP may be used to optimize outcomes of DCD livers, in particular those with no additional risk factors, although the evidence supporting HOPE use is stronger. Sequential techniques (D-HOPE-COR-NMP and NRP + MP) may be of value in DCD livers with additional risk factors, like older donor age or prolonged warm ischemia time, but studies supporting their use are still limited. In DBD donors, our meta-analysis suggests that HOPE improves graft survival, while its protective effect on IC is less evident and must be confirmed by further studies. Importantly, there is insufficient evidence to recommend any MP approach for livers with moderate or severe steatosis. Our meta-analysis did not show a significant effect of NMP on graft survival or IC incidence. The main advantage of NMP nowadays seems the possibility of assessing liver viability and enabling successful transplantation of livers that would otherwise be discarded. Additionally, although the review of studies having investigated cost-efficiency of different MP approaches^[23,105–107]^ was beyond the scope of this review, costs should be taken into due consideration, especially when two MP techniques show comparable clinical benefits.

Limitations of the present review are due to the inherent limitations of the included literature, in particular the lack of high-quality comparative studies that limits the possibility to provide practical recommendations. Its strengths, also in comparison with previous reviews on the subject, are the focus on clinically relevant endpoints, the moderator analysis investigating the influence of selected donor, recipient and logistical factors on the observed effects, and the inclusion of studies reporting on combined approaches, which might be of value in particular scenarios. By providing a broad view on the most recent literature on the subject and a critical discussion of its limitations, this review may help transplant professionals in everyday clinical decision-making about MP indications.

In conclusion, available literature suggests that each MP technique provides different advantages and that different approaches are complementary rather than mutually exclusive. However, despite a consistent and progressively growing body of literature, we are still far from defining ideal utilization scenarios for each MP technique. To fill this gap, trials focusing on well-defined donor-recipient matching and targeting relevant clinical endpoints are needed.

## Data Availability

The data, code, and other materials are available from the corresponding author upon reasonable request. The data that support the findings of this study are openly available in PubMed.

## Research registration unique identifying number (UIN)

The review protocol was registered prospectively with the National Institute for Health Research (PROSPERO) system and is available online with registration number CRD42024627523.
